# Virus-mediated EpoR76E gene therapy preserves vision in a glaucoma model by modulating neuroinflammation and decreasing oxidative stress

**DOI:** 10.1186/s12974-016-0499-5

**Published:** 2016-02-15

**Authors:** Jessica Hines-Beard, Wesley S. Bond, Jon R. Backstrom, Tonia S. Rex

**Affiliations:** Vanderbilt Eye Institute, Vanderbilt University, 11435 MRB IV, 2213 Garland Avenue, Nashville, TN 37232 USA; Vanderbilt Brain Institute, Vanderbilt University, 11435 MRB IV, 2213 Garland Avenue, Nashville, TN 37232 USA

**Keywords:** Erythropoietin, Neuroprotection, Microglia, Neuroinflammation, Glaucoma, Gene therapy, Oxidative stress

## Abstract

**Background:**

Glaucoma is a complex neurodegeneration and a leading cause of blindness worldwide. Current therapeutic strategies, which are all directed towards lowering the intraocular pressure (IOP), do not stop progression of the disease. We have demonstrated that recombinant adeno-associated virus (rAAV) gene delivery of a form of erythropoietin with attenuated erythropoietic activity (EpoR76E) can preserve retinal ganglion cells, their axons, and vision without decreasing IOP. The goal of this study was to determine if modulation of neuroinflammation or oxidative stress played a role in the neuroprotective activity of EPO.R76E.

**Methods:**

Five-month-old DBA/2J mice were treated with either rAAV.EpoR76E or a control vector and collected at 8 months of age. Neuroprotection was assessed by quantification of axon transport and visual evoked potentials. Microglia number and morphology and cytokine and chemokine levels were quantified. Message levels of oxidative stress-related proteins were assessed.

**Results:**

Axon transport and visual evoked potentials were preserved in rAAV.EpoR76E-treated mice. The number of microglia was decreased in retinas from 8-month-old rAAV.EpoR76E-treated mice, but proliferation was unaffected. The blood-retina barrier was also unaffected by treatment. Levels of some pro-inflammatory cytokines were decreased in retinas from rAAV.EpoR76E-treated mice including IL-1, IL-12, IL-13, IL-17, CCL4, and CCL5. TNFα messenger RNA (mRNA) was increased in retinas from 8-month-old mice compared to 3-month-old controls regardless of treatment. Expression of several antioxidant proteins was increased in retinas of rAAV.EpoR76E-treated 8-month-old mice.

**Conclusions:**

Treatment with rAAV.EpoR76E preserves vision in the DBA/2J model of glaucoma at least in part by decreasing infiltration of peripheral immune cells, modulating microglial reactivity, and decreasing oxidative stress.

## Background

Glaucoma is the second leading cause of blindness worldwide behind cataracts. The only modifiable risk factor for glaucoma is intraocular pressure (IOP). However, there are patients with elevated IOP who do not develop glaucoma and approximately one third of glaucoma patients have normal-range IOP. All current medical and surgical treatments focus on lowering IOP. This approach can slow the progression of disease, even in many normal-tension glaucoma patients, but often does not stop it entirely at least in part due to poor patient compliance in administering IOP lowering eye drops. As a result of the above complexities, many laboratories are focused on understanding the molecular events that underlie axon degeneration in glaucoma with the hope of intervening in the disease process in an IOP-independent manner.

Glaucoma is an axonopathy meaning that the retinal ganglion cell (RGC) axons in the optic nerve undergo pathologic changes first, followed by death of the cell bodies (for review see [1]). Several hypotheses have been posited regarding the mechanisms that induce axon degeneration in glaucoma including mechanical damage at the optic nerve head [2–5], hypoxia and oxidative stress (for reviews see [6–8]), and neuroinflammation (for review see [9, 10]). Thus, an attractive neuroprotective candidate would affect more than one of these processes. One example is erythropoietin (EPO), a type I cytokine that blocks apoptosis, preserves the blood-brain barrier, counteracts oxidative stress, and limits glial reactivity (for review see [11, 12]). Recent studies suggest that EPO can modulate neuroinflammation independently of its ability to block cell death (for review see [11]).

We previously demonstrated that recombinant adeno-associated virus (rAAV)-mediated gene delivery of EPO or EPO.R76E, a form of EPO with attenuated erythropoietic activity, prior to onset of elevated IOP preserved the RGC axons, cell bodies, and vision in the well-characterized DBA/2J mouse model of glaucoma [13]. Others have also shown that injections of EPO protein are effective in glaucoma models [14, 15]. More recently, we demonstrated that rAAV.EpoR76E is also effective in an induced model of glaucoma suggesting that the protection was not specific to the particular mouse model [16]. Further, therapeutic benefit was achieved in both models when treatment was delayed until the onset of elevated IOP although to a lesser extent than when rAAV.EpoR76E was provided earlier [16].

The goal of this study was to exploit the efficacy of EPO in blocking glaucoma pathogenesis in order to investigate the mechanisms responsible for this axonopathy in the DBA/2J model. We delivered rAAV.EpoR76E into 5-month-old mice in order to yield maximal gene expression when IOP starts to elevate in the DBA/2J [17]. We assessed therapeutic efficacy at 6 and 8 months of age, the age of onset of axon degeneration and prior to RGC death in this model [18]. We investigated the effect of EPO.R76E on neuroinflammation and oxidative stress by quantifying numbers and morphology of microglial cells, levels of pro-inflammatory chemokines and cytokines, and expression of oxidative stress-related proteins.

## Methods

### Animals

DBA/2J mice were obtained from Jackson Laboratories (Bar Harbor, ME). Mice were group-housed, maintained on a 12-h light-dark cycle, and provided food and water ad libitum. All experiments were approved by the Institutional Animal Care and Use Committee of Vanderbilt University and were in accordance with the ARVO Statement for the Use of Animals in Vision and Ophthalmic research, protocol number M/12/131. Animals were injected in the quadriceps with 1 × 10^9^ gc of rAAV2/8.CMV.EpoR76E (rAAV.EpoR76E) or rAAV2/8.CMV.eGFP (rAAV.eGFP). All viral vectors were produced by the University of Pennsylvania Vector Core (Philadelphia, PA). Mice were injected at 5 months of age and euthanized at 6 or 8 months of age.

### Fluorescein angiography (FA)

Anesthetized mice were injected with 0.1 ml of fluorescein (AK-Fluor-10 %; Akom, Inc; Lake Forest, IL). Eyes were dilated with 1 % tropicamide and 2.5 % phenylephrine and lubricated with 2.5 % methylcellulose (Goniovisc Eye Care and Cure, Inc., Tuscon, AZ). Retinal fluorescein was imaged on a Micron IV imaging system (Phoenix Research Labs, Pleasanton, CA).

### Flash visual evoked potential (fVEP)

The fVEPs were performed according to previously published methods [13, 16]. Briefly, animals were dark-adapted overnight and anesthetized with an intraperitoneal injection of ketamine/xylazine/urethane (25/6/600 mg/kg). Eyes were dilated with 1 % tropicamide and then moistened with lubricating eye drops. The fur over the visual cortex was removed, and platinum needle electrodes (Grass Technologies, Warwick, RI) were inserted subdermally over the left and right visual cortex. Electrodes were placed at the snout and flank for the reference and ground, respectively. Animals were placed on a warm platform under a Ganzfeld dome (Diagnosys LLC, Lowell, MA). Two hundred flashes of white light at an intensity of 1.0 cd · sec/m^2^ were presented to each animal at a flash frequency of 1 Hz with a 500 ms inner sweep delay. Data collection and analysis was performed by a masked investigator.

### Anterograde transport analyses

Anterograde axon transport was assessed using previously published methodology [19]. Briefly, anesthetized mice (3 % isoflurane) received single, bilateral, intravitreal injections of 2 μL of 1 % cholera toxin subunit B (CTB) conjugated to AlexaFluor-594 (Life Technologies, Carlsbad, CA) in phosphate-buffered saline (PBS) 48 h prior to collection. Brains were post-fixed in 4 % paraformaldehyde (PFA) for at least 48 h, the cortex was removed, and the remaining tissue was cryoprotected in 30 % sucrose for 48 h. Fifty-micron-thick coronal sections through the superior colliculus (SC) were obtained using a sliding microtome. Sections were mounted in Fluoromount-G (SouthernBiotech, Birmingham, AL), imaged on a Nikon Eclipse Ti epifluorescence microscope (Melville, NY), and CTB signal density in the SC was quantified using ImagePro Analyzer 7.0 software (Media Cybernetics Rockville, MD) as previously described [19]. Briefly, each SC section was divided into several bins from medial to lateral, and average CTB signal density within each bin was calculated by dividing the area of pixels above background by total pixel area measured. A colorimetric scale ranging from blue (0 % transport) to red (100 % transport) was used to indicate average CTB signal density for each bin. Each section was adjoined to the next in a rostral to caudal fashion and then oriented to generate a retinotopic map of the SC. Areas of transport deficit were subtracted from total SC area to calculate percent intact transport. Fluorescence quantification was performed by a masked investigator.

### Immunohistochemistry

For immunohistochemistry, eyes were post-fixed in 4 % PFA for at least 2 h and then whole retinas were isolated. A subset were embedded in 5 % agarose, and 70-μm-thick cross sections were collected on a vibratome. Cross sections or retinal flat mounts were incubated overnight at 4 °C in 5 % normal donkey serum in 0.1 % Triton X-100, 0.5 % BSA, 0.1 % Azide, and PBS (PBTA). Sections or flat-mounted retinas were then incubated overnight at 4 °C in primary antibody in PBTA, rinsed with PBTA, and then incubated overnight at 4 °C in secondary antibody in PBTA. Primary antibodies used: goat anti-Iba-1 (1:100; Abcam, Cambridge, UK), rabbit anti-Ki-67 (1:100; Thermo Fisher Scientific, Waltham, MA), and chicken anti-H-ferritin (1:50; Abcam, Cambridge, UK). Appropriate secondary antibodies conjugated to AlexaFluors were obtained from Life Technologies (1:200; Carlsbad, CA). Whole retinas were flat-mounted RGC side up. Both whole retinas and sections were mounted in Fluoromount-G (SouthernBiotech, Birmingham, AL), and 20-μm-thick z-stack images were collected on an Olympus FV-1000 confocal microscope (Center Valley, PA) using a ×60 oil immersion lens. Imaging on the confocal microscope was performed through the use of the Vanderbilt University Medical Center Cell Imaging Shared Resource.

The number of IBA1+ and IBA1 and Ki67+ cells were quantified in 27 high-power views of four retinas per treatment condition. A total of 207 and 168 IBA-1 positive cells were assessed from retinas of mice injected with rAAV.eGFP or rAAV.EpoR76E, respectively. Immunofluorescence of H-ferritin was quantified according to previously published methods [20]. Briefly, ×60 magnification confocal micrographs at a 10-μm depth within the tissue were used for quantification. Fluorescence intensity from the ganglion cell layer to the outer plexiform layer was quantified and averaged using ImageJ software. The intensity was normalized based on total area of retina assessed.

### Microglial quantification and morphometrics

Flat-mounted retinas immunolabeled with Iba-1 were imaged through the inner plexiform layer (IPL) beginning just below the RGC layer in five central and five peripheral areas. All microglial analyses were conducted by a masked investigator using ImagePro Analyzer 7.0 software. In each z-stack image, microglia number and soma area were quantified. Ramification number for each microglial cell was determined by counting intersections of glial processes with a 10 × 10 μm grid overlay.

### Multiplex ELISA

Retinas were sonicated and run in singlet on the Milliplex MAP Mouse Cytokine/Chemokine Magnetic Bead Panel I 25-plex assay per manufacturer’s protocol (EMD Millipore, Billerica, MA). Plates were read on a Luminex MAGPIX with xPONENT software (Thermo Fisher Scientific, Waltham, MA) using settings stated in the manufacturer’s protocol. Multiplex ELISA plates were read and analyzed through the use of the Vanderbilt Hormone Assay and Analytical Services Core.

### Quantitative reverse transcription-PCR

Retinas were homogenized, and RNA was extracted using a Qiagen RNeasy kit (Valencia, CA). RNA concentration and purity was measured on a spectrophotometer. A first-strand complementary DNA (cDNA) library was synthesized from 250 ng of RNA from each sample using the Superscript III First-Strand synthesis system (Invitrogen, Waltham, MA) and oligo-dT_20_ primers. Quantitative PCR (qPCR) was performed using Power SYBR green master mix (Applied Biosystems, Waltham, MA). All primer sequences were obtained from previous studies; we assessed the following: iNOS, arginase 1, IL-1β, IL-6, TNFα, TGFβ, IL-4, and IL-10 [21–24]. All qPCR was performed in duplicate using the Applied Biosciences 7300 real-time PCR system (Waltham, MA). The assay was performed in duplicate on 13 retinas per condition. The amplification threshold was set using system software. Relative changes in gene expression were determined using glyceraldehyde-3-phosphate dehydrogenase (GAPDH) as an internal control [25]. There was no significant change between samples in GAPDH levels.

### Oxidative stress PCR array

The same cDNA as was used for the quantitative PCR analysis was also used in an RT2 Profiler mouse oxidative stress PCR array kit per manufacturer’s instructions (Qiagen, Valencia, CA). The cDNA from each group was pooled to have sufficient material for the assay.

### Statistical analysis

All statistical analyses were performed using GraphPad Prism software (La Jolla, CA). A two-way ANOVA with a Bonferroni post hoc test was performed for the hematocrit time course. A one-way ANOVA with a Bonferroni post hoc test (*α* = 0.05) was used to analyze anterograde transport data, microglia morphometric data, ON quantification data, and fVEP latencies. Percent baseline fVEP amplitudes and results from the multiplex ELISA were compared using an unpaired Student’s *t* test (*α* = 0.05). A one-way ANOVA and Dunnett’s multiple comparisons post hoc test (*α* = 0.05) were used to analyze the qPCR results. Means and standard deviation were calculated for each data set.

## Results

### Delayed treatment with rAAV.EpoR76E protects against vision loss in the DBA/2J model of glaucoma

**Fig. 1:**
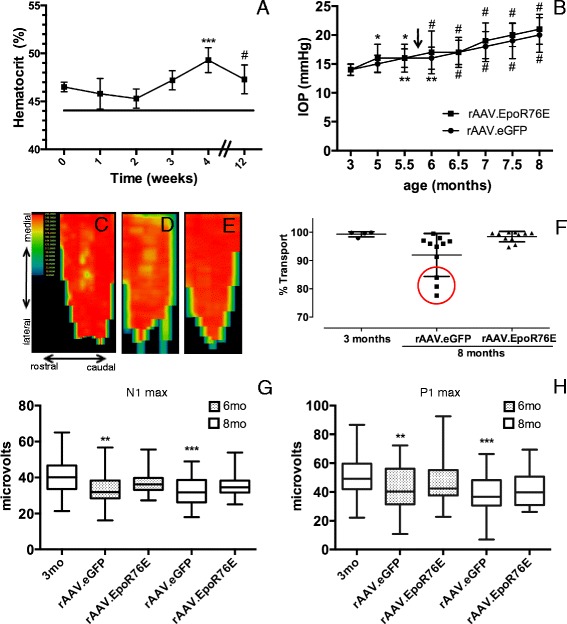
Treatment with rAAV.EpoR76E at 5 months protects against vision loss at 6 and 8 months of age. **a** Graph of hematocrit level over time in mice after systemic injection of rAAV.EpoR76E compared to baseline levels in rAAV.eGFP-injected mice (*line*) showing a statistically significant increase at 4 and 12 weeks. **b** Graph of IOP level over time showing a statistically significant increase by 5.5 months of age in both rAAV.eGFP- and rAAV.EpoR76E-treated mice as compared to levels at 3 months of age. There was no difference between treatment groups at any time-point. *Arrow* indicates when an increase in hematocrit was first detected after gene delivery of EpoR76E. **c**–**e** Representative SC fluorescence heat maps of (**c**) 3-month-old mice, (**d**) 8-month-old mice treated with rAAV.eGFP, and (**e**) 8-month-old mice treated with rAAV.EpoR76E. Orientation labels in (**c**) apply to all heat maps. **f** Scatter plot of percent axon transport to the SC. The *red circle* indicates optic nerves with poor axon transport, note the lack of nerves with poor transport in the rAAV.EpoR76E-treated cohort, *p* < 0.0001 by Bartlett’s test. **g** *Box* and *whisker plots* of fVEP N1 peak amplitude shown as absolute values. **h** *Box* and *whisker plots* of fVEP P1 peak amplitude. The amplitudes in the rAAV.eGFP-treated mice were decreased as compared to 3-month-old controls. There was no statistically significant difference between the rAAV.EpoR76E-treated mice and either other group. *p < 0.05, **p < 0.01, ***p < 0.001, and ^#^ *p* < 0.0001

Maximal protein production from rAAV2/8-mediated transgene delivery after an intramuscular injection is typically achieved 3 weeks after injection [17]. Since EPO.R76E causes a mild elevation in hematocrit, we used that as a physiological read-out of EPO.R76E production. There was no statistically significant difference in hematocrit levels in rAAV.EpoR76E-injected mice as compared to averaged baseline/rAAV.eGFP-injected mice (see line, Fig. 1a) until 4 weeks after gene delivery (*p* < 0.01; Fig. 1a). This elevation was retained for the duration of the study (12 weeks). While the increase in hematocrit at 3 weeks was not statistically significant, the average was the same as that detected at 12 weeks (47 %, as compared to 44 % in the rAAV.eGFP group). The only difference is that we only have an “n” of 3 at 3 weeks but an “n” of 23 at 12 weeks. Since the elevation in hematocrit is so slight, it was not sufficient to result in a statistical significance at 3 weeks. For this reason, and the fact that hematocrit is a physiological read-out rather than a direct measure of EPO.R76E levels, our data is still consistent with an onset of significant transgene expression at 3 weeks after injection. Since mice were injected at 5 months of age, the increase in EPO.R76E correlates to 5.75 months of age (arrow, Fig. 1b). The average increase in hematocrit was comparable to our previous results with rAAV.EpoR76E (Fig. 1a) [13, 26, 27]. We have previously demonstrated that EPO.R76E enters the eye after systemic rAAV gene delivery [16, 28]. The elevation in IOP was variable, consistent with this model, but reached statistical significance compared to 3 months in both groups by 5.5 months of age (Fig. 1b). Consistent with our previous studies, there was no difference in IOP elevation between mice that received rAAV.eGFP and those that received rAAV.EpoR76E (Fig. 1b) [13, 16].

Active anterograde axon transport from the RGC to the SC was quantified according to previously published methods to assess axon integrity [16, 19]. The heat map of fluorescence intensity shows intact axon transport in the 3-month-old DBA/2J mice (Fig. 1c). There was decreased axon transport particularly along the caudal edge of the SC from 8-month-old DBA/2J mice treated with rAAV.eGFP (Fig. 1d). In contrast, axon transport appeared completely preserved in the 8-month-old DBA/2J mice that received rAAV.EpoR76E (Fig. 1e). The fluorescence in the SC was quantified to determine the percent intact transport (Fig. 1f). The 3-month-old mice exhibited 99 ± 0.9 % (avg ± s.d., *n* = 4) intact transport. Consistent with the original characterization of the DBA/2J, axon transport deficits were just beginning to occur at 8 months of age 92 ± 7.6 % (*n* = 11) with two populations of mice clearly evident and statistically significant by Bartlett’s test of variance, *p* < 0.0001. At this early time-point, only 27 % of the axons exhibited axon transport levels similar to the 3-month control, while 45 % had axon transport levels below 95 % (lowest was 78 %). In contrast, 83 % of the axons from mice treated with rAAV.EpoR76E had a percent axon transport of greater than 95 % (*n* = 11).

Consistent with our results in 10-month-old DBA/2J mice [13, 16], there was no effect of glaucoma or rAAV.EpoR76E on the fVEP N1 or P1 latency at 6 or 8 months of age (data not shown). There was a statistically significant decrease in both the N1 (Fig. 1g) and P1 (Fig. 1h) amplitudes at 6 (*p* < 0.01) and 8 months (*p* < 0.001) of age in mice injected with rAAV.eGFP as compared to 3-month-old DBA/2J controls. The N1 and P1 amplitudes in mice treated with rAAV.EpoR76E were not different from 3-month-old DBA/2J mice or rAAV.eGFP-injected mice suggesting a moderate protective effect by rAAV.EpoR76E.

### Microglia number, but not proliferation, was significantly decreased by rAAV.EpoR76E

In order to assess a role for neuroinflammation, we examined relevant molecular and cellular changes at 8 months, a time-point prior to cell death and at the beginning stages of axon degeneration in the DBA/2J [18]. Increases in microglial number and reactivity are detected as early as 3 months of age, prior to the onset of elevated IOP in this model [29]. The alteration in microglia occurs first in the central retina and expands to the peripheral retina over time [29]; thus, we report changes in the central and peripheral retina separately.

**Fig. 2:**
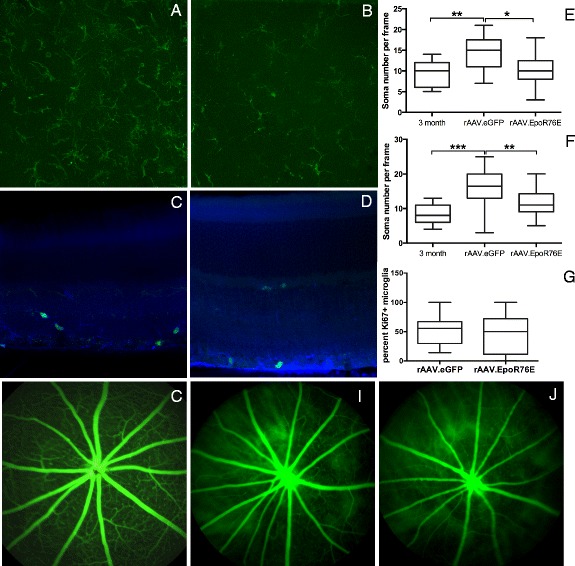
Treatment with rAAV.EpoR76E reduced the number, but not proliferation, of microglia in the retina. **a**, **b** Representative confocal micrographs of IBA-1 immunolabeling (*green*) of flat-mounted retinas from 8-month-old mice treated with rAAV.eGFP (**a**) or rAAV.EpoR76E (**b**). **c**, **d** Representative confocal micrographs of anti-IBA-1 (*blue*) and anti-Ki67 (*green*) double-labeling in retinal sections from 8-month-old mice treated with rAAV.eGFP (**c**) or rAAV.EpoR76E (**d**). **e**, **f** *Box* and *whisker plots* showing quantification of microglia number in the central (**e**) and peripheral (**f**) retinas. *p < 0.05, **p < 0.01, and ***p < 0.001. **g** *Box* and *whisker plots* of the percentage of anti-IBA-1-positive cells that were also anti-Ki67-positive. There was no statistically significant difference between rAAV.eGFP- and rAAV.EpoR76E-treated mice. **h**–**j** Representative fluorescein angiography in a normal retina (**h**) and glaucomatous retinas from mice treated with rAAV.eGFP (**i**) or rAAV.EpoR76E (**j**)

Microglia in the inner plexiform layer of the retina appeared increased in the 8-month-old DBA/2J mice injected with rAAV.eGFP as compared to those treated with rAAV.EpoR76E (Fig. 2a, b). Quantification yielded an average number of IBA-1-positive microglia in the central retinas of 3-month-old mice and 8-month-old rAAV.eGFP- and rAAV.EpoR76E-treated mice of 9.3 ± 3.4, *n* = 15; 14 ± 4.2, *n* = 25 (*p* < 0.01 vs 3 month); and 10 ± 4.1, *n* = 30 (n.s. vs 3 month), respectively (Fig. 2e). The difference in the number of microglia in the central retinas of rAAV.eGFP- and rAAV.EpoR76E-treated mice was also statistically significant, *p* < 0.05. The average number of microglia in regions of the peripheral retina of 3-month-old mice and 8-month-old rAAV.eGFP- and rAAV.EpoR76E-treated mice was 8.7 ± 2.7, *n* = 15; 16 ± 5.5, *n* = 24 (*p* < 0.001 vs 3 month); and 12 ± 3.8, *n* = 30 (n.s. vs 3 month), respectively (Fig. 2f). The difference in the number of microglia in the peripheral retinas of mice treated with rAAV.eGFP or rAAV.EpoR76E-treated mice was also statistically significant, *p* < 0.01.

To determine if the decrease in microglia was due to inhibition of microglial proliferation, we performed double immunolabeling with the microglial marker, anti-IBA1, and anti-Ki67, a marker for proliferating cells. Double-labeled cells were evident in 8-month-old retinas treated with either rAAV.eGFP (Fig. 2c) or rAAV.EpoR76E (Fig. 2d). An average of 51 ± 4.2 % (SEM) of IBA-1-positive microglia were Ki67-positive in retinas from rAAV.eGFP-injected 8-month-old mice (Fig. 2g). Similarly, in retinas from rAAV.EpoR76E-treated mice, 44 ± 6.9 % of IBA-1-positive microglia were Ki67-positive.

The retinal vasculature was assessed by fluorescein angiography to determine if the decrease in number of microglial cells was due to preservation of the blood-retina barrier by EPO.R76E. In the normal, non-glaucomatous, retina, fluorescein is retained within the blood vessels (Fig. 2h). However, in the glaucomatous DBA/2J, retina leakage of fluorescein was detected regardless of treatment (Fig. 2i, j).

### EPO.R76E-modulated microglial morphology in 8-month-old DBA/2J mice

**Fig. 3:**
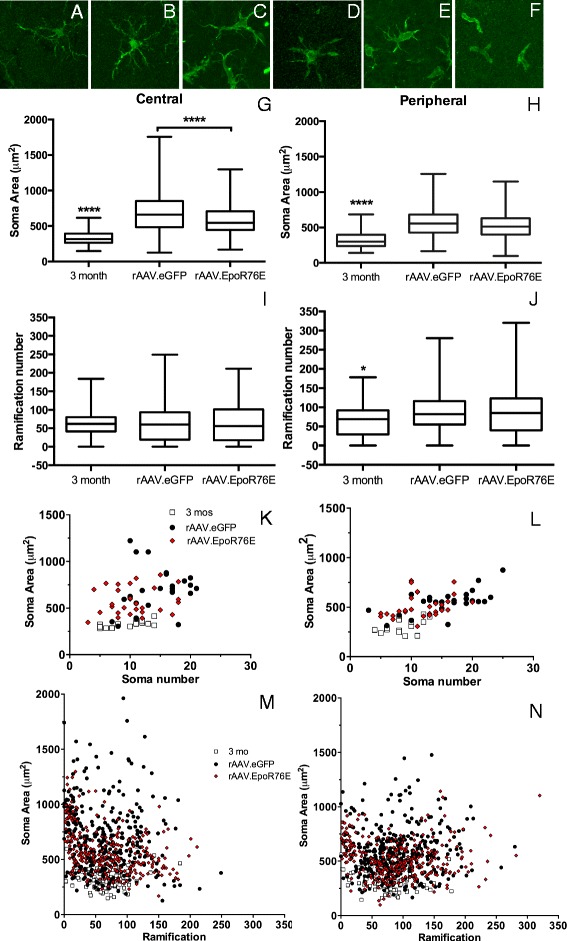
Treatment with rAAV.EpoR76E altered microglial morphology. **a**–**f** Representative high-magnification confocal micrographs of IBA-1 immunolabeled (*green*) microglia in 3-month-old (**a**) and 8-month-old (**b**–**f**) retinas. These morphologies were present in retinas from both treatment groups. **g**, **h** *Box* and *whisker plots* of microglial soma area of cells from the central (**g**) or peripheral (**h**) retina, ****p < 0.0001. **i**, **j** *Box* and *whisker plots* of microglial ramification number for cells from the central (**i**) or peripheral (**j**) retina, *p < 0.05. **k**, **l** *Scatter graphs* of microglial soma area versus soma number in the central (**k**) or peripheral (**l**) retina. **m**, **n** *Scatter graphs* of microglial soma area versus ramification number in the central (**m**) or peripheral (**n**) retina

To determine if rAAV.EpoR76E altered the reactive state of the microglia, the morphology of the cells was assessed both in the central and peripheral retina. In 3-month-old control retinas, most microglial cells had small somas and highly stratified, thin processes (Fig. 3a). Regardless of treatment condition, a wide range of microglial morphologies were detected in glaucomatous retinas including larger cell bodies and shortened processes (Fig. 3b–f). The ranges of microglial morphologies are shown, starting with thicker processes and ending with amoeboid morphologies. Examples of all stages were detected in retinas from both rAAV.eGFP- and rAAV.EpoR76E-injected 8-month-old DBA/2J mice.

The average soma area in the central and peripheral regions of retinas from 3-month-old mice was 328 ± 93 μm^2^ (*n* = 139 cells) and 320 ± 108 μm^2^ (*n* = 131 cells), respectively (Fig. 3g, h). These values were statistically different from the soma size in retinas from 8-month-old mice regardless of treatment, *p* < 0.0001. In addition, there was a statistically significant difference in soma area in the central retinas of the rAAV.eGFP (698 ± 296 μm^2^, *n* = 357 cells)- and rAAV.EpoR76E (598 ± 221 μm^2^, *n* = 289 cells)-treated 8-month-old mice, *p* < 0.0001 (Fig. 3g). In contrast, the difference between the treatment groups in the peripheral retina at 8 months was not statistically significant. We detected 578 ± 204 μm^2^ (*n* = 394 cells) and 535 ± 184 μm^2^ (*n* = 312) in retinas from rAAV.eGFP- and rAAV.EpoR76E-treated mice, respectively (Fig. 3h). As expected, the soma area was larger in both 8-month groups in the central retina as compared to peripheral retina, matching a previous study [29].

The average number of microglial ramifications in the 3-month central and peripheral retina was 61 ± 32 (*n* = 121 cells) and 63 ± 43 (*n* = 103 cells), respectively (Fig. 3i, j). The microglia in the central retinas of the 8-month-old DBA/2J mice had a similar number of ramifications regardless of treatment, 62 ± 47 (*n* = 329 cells) and 64 ± 50 (*n* = 285 cells) in the rAAV.eGFP- and rAAV.EpoR76E-treated mice, respectively (Fig. 3i). In contrast, more ramifications were detected in the 8-month-old peripheral retina compared to the central retina regardless of treatment (*p* < 0.001). A similar number of microglial ramifications were detected in cells from the peripheral retina; there was an average of 88 ± 48 (*n* = 367 cells) ramifications in the rAAV.eGFP-treated mice and 87 ± 59 (*n* = 310 cells) in the rAAV.EpoR76E-treated mice (Fig. 3j).

By plotting the soma area against soma number, the moderate effect of rAAV.EpoR76E on microglia morphology becomes more apparent (Fig. 3k, l). The microglia from 3-month-old retinas are primarily clumped together with a soma area under 500 μm^2^ and a ramification number of under 100. In contrast, there was significant variability in the morphology of the microglia from retinas of 8-month-old rAAV.eGFP-treated mice. The microglial response in the 8-month-old retinas of rAAV.EpoR76E-treated mice was more restricted than the rAAV.eGFP-treated mice, but the soma size and ramification numbers were still elevated as compared to the 3-month controls. Similarly, by plotting soma area against ramification number, it is again apparent that treatment with rAAV.EpoR76E dampens the reactivity of the microglia but does not restore them entirely to a quiescent state (Fig. 3m, n).

### Retinas from rAAV.EpoR76E-treated mice had lower levels of pro-inflammatory cytokines/chemokines

**Fig. 4:**
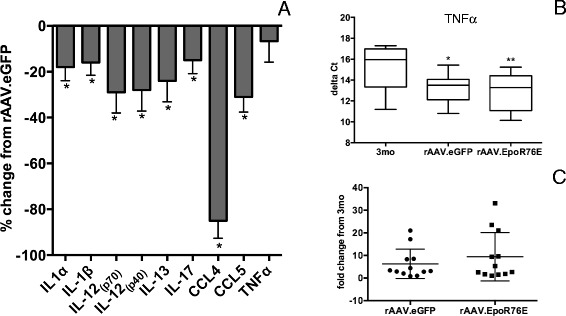
Treatment with rAAV.EpoR76E causes a decrease in pro-inflammatory cytokines and chemokines. **a** *Bar graph* of the percent decrease in pro-inflammatory cytokines in retinas from 8-month-old rAAV.EpoR76E-treated mice as compared to mice treated with rAAV.eGFP, *p < 0.05. **b**, **c** TNFα mRNA levels are increased in glaucoma and unaffected by treatment with rAAV.EpoR76E. *Box* and *whisker plots* of the delta Ct (**b**) and fold change (**c**) in TNFα mRNA in 3-month-old and 8-month-old mice treated with rAAV.EpoR76E or rAAV.eGFP, *p < 0.05

Another measure of neuroinflammation is the level of cytokines and chemokines that are produced by reactive glia (microglia, astrocytes, and Müller cells). We detected statistically significant (*p* < 0.05) decreases in some pro-inflammatory cytokines (Fig. 4a). As compared to rAAV.eGFP, we detected 18, 16, 15, 28, 29, 85, 31, and 24 decreases in IL-1α, IL-1β, IL-17, IL-12_p40_, IL-12_p70_, CCL4 (MIP-1β), CCL5 (RANTES), and IL-13, respectively, in retinas from mice treated with rAAV.EpoR76E. There were no statistically significant differences between the rAAV.eGFP and rAAV.EpoR76E retina levels of other pro-inflammatory cytokines/chemokines: TNFα, IL-6, CCL2, CXCL10, IL-2, IL-7, MIP 2, IFNγ, GM-CSF, IL-10, IL-5, G-CSF, IL-9, CXCL1, CCL3 (MIP1α), and IL-4 (data not shown).

We also measured messenger RNA (mRNA) levels for markers of reactive microglia. There was no change detected in levels of IL-1β, iNOS, arginase-1, IL-4, IL-6, IL-10, or TGFβ message compared to 3-month controls or between treatment groups. In contrast, there was a statistically significant increase in mRNA for TNFα in the rAAV.eGFP group (*p* < 0.05) and rAAV.EpoR76E (*p* < 0.01) retinas as compared to the 3-month controls (Fig. 4b). This calculated to a fold increase in TNFα mRNA of 6.3 ± 1.9 (avg ± SEM) and 9.4 ± 3.1 in rAAV.eGFP- and rAAV.EpoR76E-treated mice, respectively, as compared to 3-month controls (Fig. 4c).

### Retinas from rAAV.EpoR76E-treated mice had increased levels of antioxidant enzymes

**Fig. 5:**
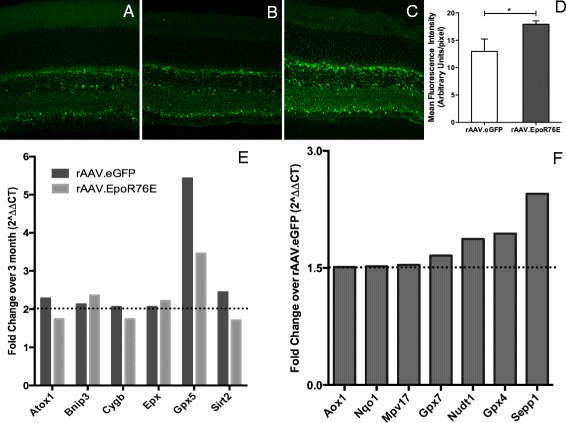
Treatment with rAAV.EpoR76E increases expression of several antioxidant proteins and increases H-ferritin in the retina. **a**–**c** Representative confocal micrographs of H-ferritin immunolabeling in retinas from 3-month controls (**a**), 8-month-old mice injected with rAAV.eGFP (**b**), and 8-month-old mice treated with rAAV.EpoR76E (**c**). **d** *Bar graph quantification* of H-ferritin immunofluorescence showing a statistically significant increase in the rAAV.EpoR76E-treated group as compared to rAAV.eGFP-injected mice. **e** *Bar graph* of mRNAs with increased expression in 8-month glaucomatous retina as compared to 3-month controls regardless of treatment. **f** *Bar graph* of mRNAs with increased expression in retinas from rAAV.EpoR76E-treated 8-month-old mice as compared to age-matched rAAV.eGFP-injected mice

Glaucoma pathogenesis is associated with neuroinflammation, hypoxia, and mitochondrial dysfunction, all of which result in oxidative stress [8–10, 30]. EPO.R76E, although to a lesser extent than wild-type EPO, does increase erythropoiesis and therefore may increase oxygenation to the retina. To test this hypothesis, we performed immunolabeling for H-ferritin, a sensitive measure of tissue iron levels (Fig. 5a–c) [31]. Tissue H-ferritin is increased when hepcidin levels are reduced as a result of increased erythropoiesis [32, 33]. The labeling pattern was unchanged between groups; however, quantification of inner retina fluorescence showed an increase in the retinas from rAAV.EpoR76E-treated mice as compared to those injected with rAAV.eGFP (Fig. 5d).

Further, EPO can activate NF-E2-related factor 2 (Nrf2) to induce gene expression from the antioxidant response element (ARE) [34]. Therefore, we investigated if EPO.R76E altered expression levels of oxidative stress-related proteins in the glaucomatous DBA/2J mouse retina at 8 months (Fig. 5e, f). A twofold or greater increase was detected in BCL2/adenovirus E1B 19 kDa interacting protein 3 (Bnip3), eosinophil peroxidase (Epx), and glutathione peroxidase 5 (Gpx5) as compared to 3-month-old controls regardless of treatment (Fig. 5e). Expression of Bnip3 is induced by hypoxia inducible factor 1α (HIF-1 α); the protein is localized to the mitochondrial outer membrane where it can both induce mitophagy of mitochondria and activate apoptosis (for review see [35]). Increases in Gpx5 have been reported in glaucoma patients [36, 37]. Levels of antioxidant 1 copper chaperone (Atox1), cytoglobin (Cygb), and sirtuin 2 (Sirt2) were elevated 1.5-fold in both groups as compared to 3-month controls (Fig. 5e). ATOX1 is an antioxidant against superoxide and hydrogen peroxide. CYGB is expressed in retinal neurons where it can bind oxygen to prevent oxidative stress. Altogether, the increase in expression of these enzymes suggests that the retina enacts an endogenous protective response against oxidative stress early in glaucoma.

There was an increase of 1.5-fold or greater in aldehyde oxidase 1 (Aox1) also known as retinal oxidase [38, 39], NADPH quinone oxidoreductase 1 (Nqo1), the mitochondrial inner membrane protein (Mpv17), Gpx7, nucleoside diphosphate-linked moiety X-type motif 1 (Nudt1), Gpx4, and selenoprotein P plasma 1 (Sepp1) in the retinas from rAAV.EpoR76E as compared to rAAV.eGFP-treated 8-month-old glaucomatous mice (Fig. 5f). The increase in Aox1 was a surprise as it produces hydrogen peroxide and can also catalyze the formation of superoxide. The remaining mRNAs encode antioxidant enzymes, several of which are associated with the mitochondria (Nqo1, Nudt1, and Gpx). Sepp1 is interesting in that it encodes an extracellular glycoprotein that has an antioxidant role and appears to be associated with endothelial cells [40].

## Discussion

We detected a deficit in vision at 6 months of age, prior to a quantitative decrease in axon transport or axon degeneration, suggesting that the fVEP may be the earliest indicator of disease progression in this well-recognized progressive model of pigment dispersion glaucoma. The time course assessed in this study also revealed that delivery of rAAV.EpoR76E at the earliest sign of elevated IOP (5 months) preserved axon transport as soon as 1 month later. The serotype of rAAV that we used in this study results in gene expression approximately 3 weeks after injection [17], so it is impressive that preservation of vision was already detectable at 6-months, after only 1 week of peak gene expression. This treatment effect is also long-lasting [13, 16], making it ideal for glaucoma, a slow, progressive disease. Finally, treatment with EPO.R76E is safer than wild-type EPO because it does not cause a dangerous rise in hematocrit [13, 26, 27, 41].

The blood-retina barrier is disrupted both in patients and in the DBA/2J model [42–44], and the DBA/2J mouse demonstrates enhanced transendothelial monocyte migration into the optic nerve head that contributes to disease pathogenesis [44]. Further, others have shown that EPO preserves the blood-brain barrier in models of brain injury by blocking endothelial cell death and astrocyte hypertrophy (for review see [11]), and we have previously shown that EPO.R76E retains these activities in in vitro assays [28, 45]. Despite this, the blood-retina barrier appeared equally disrupted in both rAAV.eGFP- and rAAV.EpoR76E-treated groups in this study. Thus, the decrease in number of microglial cells by rAAV.EpoR76E was neither due to inhibition of proliferation nor from preservation of the blood-retina barrier. Rather, our results show that treatment with rAAV.EpoR76E resulted in lower levels of several molecules known to promote the recruitment and infiltration of Th1 cells from the periphery: IL-12p40 (part of IL-23) [46], CCL4, and CCL5 (for review see [47]), with the decrease in CCL4 being particularly dramatic.

In addition to decreasing the number of microglial cells, treatment with rAAV.EpoR76E modulated the microglial response to glaucoma in the DBA/2J. EPO is anti-apoptotic and thus could limit neuroinflammation by blocking cell death (for review see [12]). However, this is unlikely to explain our results since the analyses were performed at a time-point prior to the onset of significant RGC death [18]. The decrease in levels of pro-inflammatory cytokines/chemokines in retinas from rAAV.EpoR76E-treated mice could be due to the presence of fewer microglia. Alternatively, it could reflect decreased production from macroglial cells (astrocytes and Müller cells) or the remaining microglial cells. The morphometric analysis shows that treatment with rAAV.EpoR76E did not completely block microglial reactivity. We posit that this is an advantage of EPO therapy since in other models, blocking the microglial response entirely can worsen neurodegeneration while modulating it is neuroprotective (for review see [48]).

The lack of difference in TNFα levels between the two treatment groups was unexpected considering EPO has been reported to decrease levels of TNFα in the lipopolysaccharide model of ocular inflammation [49, 50]. It is possible that glaucoma might induce TNFα production through a different signaling pathway than that activated by lipopolysaccharide [49, 50]. The lack of correlation between microglia number and TNFα levels suggests that the majority of TNFα in the glaucomatous retina may be produced by reactive macroglia rather than microglia. This is supported by a previous study that demonstrated production of TNFα from primary retinal astrocytes and Müller cells under stress conditions [51]. This, in turn, may suggest that systemic gene delivery of EPO.R76E had little to no effect on the reactivity of these macroglial cells. We have previously demonstrated decreased glial reactivity after intraocular, but not systemic, delivery of EPO suggesting a possible dose-dependent effect (our unpublished data) [45]. In addition, the detection of neuroprotection by EPO.R76E despite a lack of decrease in TNFα levels suggests that EPO.R76E acts downstream or independent of the TNFα pathway.

TNFα can activate the caspase cell death cascade, induce mitochondrial dysfunction, and cause oxidative damage [51, 52]. Since the current study was performed prior to initiation of cell death in this model, that mechanism is less relevant [18]. EPO can decrease oxidative stress, at least in part, through activation of Nrf-2 and the ARE (for review see [11]) [34]. We detected increased expression of several antioxidant enzymes and proteins in the retinas of rAAV.EpoR76E-treated mice including those involved in mitochondrial function (Nqo1, Nudt1, Gpx). Therefore, independent of modulation of neuroinflammation, EPO.R76E may protect against glaucomatous neurodegeneration by directly counteracting oxidative stress. If this is the major pathway for neuroprotection by EPO.R76E, then increasing expression of Nrf2 may be just as effective. In support of this approach, others have shown that chemically induced activation of Nrf2 protects RGCs in the optic nerve crush model of RGC death [53].

Finally, hypoxia has been implicated as a major contributor to glaucoma pathogenesis [8]. The hypothesis states that damage to the microvasculature at the optic nerve head leads to hypoxic events. This hypothesis is supported by studies showing an increase in levels of HIF1α in the vitreous of glaucoma patients and in animal models [54, 55]. A major target of HIF1α is EPO and both hypoxic pre-conditioning and systemic treatment with EPO are protective to retinal neurons [56, 57]. EPO expression is also increased in glaucoma patients and in a rat model of ocular hypertension suggesting that it may represent an endogenous protective mechanism [58–62]. Systemic exogenous EPO can increase tissue oxygen delivery as a result of increased erythropoiesis. We detected an increase in H-ferritin immunofluorescence in the retinas of rAAV.EpoR76E-treated mice suggesting that although erythropoiesis was significantly attenuated as compared to that induced by wild-type EPO, it was still sufficient to increase blood flow to the retina.

## Conclusions

The current results support findings from other groups that neuroinflammation, hypoxia, and oxidative stress play a role in glaucoma pathogenesis (for review see [8–10, 30]). We expect that the most successful neuroprotective approach for a complex neurodegenerative disease such as glaucoma will require blocking neuronal cell death as well as normalizing the environment, including restoring appropriate glial support. We have shown that EPO.R76E is a very promising therapeutic to accomplish this goal.
